# Synergistic Regulation of Waste Glass Powder and Aggregate Packing Structure for Sustainable Cement Mortar: Microstructural Mechanisms and XGBoost-PSO Intelligent Mix Design Optimization

**DOI:** 10.3390/ma19194202

**Published:** 2026-10-01

**Authors:** Yanfeng Wang, Yongsen Yang, Keqing Hu, Jiahong Zhang, Yi Zhao, Qingli Lin

**Affiliations:** 1 School of Intelligent Construction and Building Engineering, Zhongyuan University of Technology, Zhengzhou 450007, China; 2 Key Laboratory of Engineering Materials of Ministry of Water Resources, China Institute of Water Resources and Hydropower Research, Beijing 100038, China; yangys@iwhr.com (Y.Y.);

**Keywords:** waste glass powder, cement mortar, aggregate packing, microstructure, machine learning, XGBoost-PSO optimization

## Abstract

**Highlights:**

• XGBoost-PSO enables intelligent optimization of sustainable mortar mixtures.
• XGBoost accurately predicts compressive and flexural strength under coupled mixture-design variables, outperforming LR, SVR, and RF.
• SEM and MIP reveal the microstructural origin of mechanical enhancement.
• Moderate WGP incorporation improves strength through pore refinement and pozzolanic reaction.
• Aggregate packing governs the effectiveness of waste glass powder in cement mortar.

**Abstract:**

Waste glass powder (WGP) is a promising recycled constituent for sustainable cementitious materials, yet its effectiveness depends strongly on the packing characteristics of the fine-aggregate skeleton. This study investigates the coupled effects of WGP replacement ratio and fine-aggregate gradation on the mechanical performance and microstructural evolution of cement mortar. Five aggregate gradations and four WGP replacement levels (0%, 8%, 10%, and 12% by mass) were evaluated through compressive and flexural strength tests at 7, 14, and 28 days, yielding 180 prism specimens in total. Scanning electron microscopy (SEM) and mercury intrusion porosimetry (MIP) were used to identify the governing microstructural mechanisms. Four regression models—linear regression (LR), support vector regression (SVR), random forest (RF), and extreme gradient boosting (XGBoost)—were compared for compressive strength prediction under identical data partitioning. XGBoost achieved the highest accuracy (R^2^ = 0.9346, RMSE = 0.9860 MPa, MAE = 0.6943 MPa), outperforming RF (R^2^ = 0.8896), LR (R^2^ = 0.8845), and SVR (R^2^ = 0.8612), and was subsequently coupled with particle swarm optimization (PSO) for constrained mixture design. Appropriate aggregate packing substantially improved WGP utilization by combining micro-filling, secondary pozzolanic reaction, pore refinement, and interfacial transition-zone densification. The best experimental combination (GCS10-M3) increased 28-day compressive and flexural strengths by 14.0% and 42.9%, respectively, relative to the reference mortar with the same gradation. The XGBoost-PSO framework identified a theoretical optimum containing 14.86% WGP, beyond the tested range, in a fines-rich bimodal aggregate gradation, with predicted 28-day compressive and flexural strengths of 41.10 MPa and 8.23 MPa, respectively. The results establish a mechanism-informed, data-driven route for designing resource-efficient mortar for building engineering applications.

## 1. Introduction

The construction industry is under increasing pressure to reduce its environmental footprint. Ordinary Portland cement (OPC) production is among the most carbon-intensive industrial processes globally, accounting for approximately 5–8% of anthropogenic CO_2_ emissions, and its manufacture consumes substantial quantities of limestone, clay, and thermal energy [1]. Against this backdrop, the partial replacement of cement or natural fine aggregate with post-consumer wastes and industrial by-products has become a principal strategy for circular and sustainable building materials [2,3]. Among available waste streams, waste glass powder (WGP) is particularly attractive because cullet is generated in large volumes worldwide and contains a high proportion of amorphous SiO_2_—a composition that confers both physical and, under appropriate conditions, chemical functionality in cementitious matrices.

In the present study, WGP is used primarily as a fine-aggregate replacement, substituting the corresponding size fractions of natural sand on a mass basis. This substitution mode is distinct from supplementary cementitious material (SCM) cement replacement, and the governing mechanism at the macro-scale is physical: WGP particles fill interstitial voids within the aggregate skeleton and modify packing geometry. Where WGP is sufficiently fine and the local alkaline environment is adequate, a secondary pozzolanic reaction may occur—the reactive SiO_2_ reacts with portlandite (Ca(OH)_2_) released during cement hydration to generate additional calcium silicate hydrate (C-S-H) [4,5]. This secondary chemical contribution is real but context dependent, and it should be understood as a modifier of the primary physical role rather than the defining mechanism of the material system studied here.

A substantial body of prior research has examined WGP in cementitious materials, and the findings are broadly encouraging but inconsistent on key quantitative parameters. Ishaq et al. [4] reported that 10–15% WGP as cement replacement densified the paste microstructure through combined pozzolanic and filler action in binary concrete systems. Al-Sodani [5] demonstrated that hybrid waste brick powder–glass powder systems retained adequate compressive strength and improved microstructural continuity after exposure to elevated temperatures. Balasubramanian et al. [6] partially replaced both cement and e-plastic with WGP and confirmed compressive strength improvement up to an optimum dosage, beyond which performance declined. Ibrahim [7] found that WGP in ternary blends with silica fume and fly ash achieved compressive strength gains up to 20% at 15% substitution, attributing the improvement to synergistic SCM densification. Mohammed and Hama [8] observed that WGP improved both mechanical strength and impact resistance of green concrete, with flexural strength showing a proportionally larger gain than compressive strength owing to strengthened interfacial transition zones (ITZs). Jain et al. [9] evaluated the durability performance of granite–glass powder concrete and reported improved chloride resistance alongside strength gains. Jani and Hogland [10] conducted an extensive review of glass waste in cement and concrete production and identified particle fineness as the primary determinant of pozzolanic efficiency, with coarser WGP acting predominantly as an inert filler. Zhan et al. [11] studied recycled aggregate concrete incorporating WGP and steel slag powder and reported reductions in total porosity and improvements in nanomechanical properties at ITZs. Muhedin and Ibrahim [2] demonstrated that WGP can replace both cement and sand fractions without structural penalty, while Baikerikar et al. [3] confirmed environmental and cost benefits of combined glass powder–glass sand substitution in eco-friendly concrete. Li et al. [12] observed that WGP dosages up to 10% improved pervious concrete compressive strength, with performance declining at higher dosages due to matrix dilution. Despite these advances, reported optimal WGP replacement levels span 5–25% across studies, a variability commonly attributed to differences in particle fineness, substitution mode, water-to-binder ratio, curing conditions, and matrix composition [13]. A factor that has received comparatively little systematic attention is the particle-size distribution of the fine-aggregate skeleton. Aggregate gradation governs initial packing density, interstitial-void distribution, paste demand, and spatial conditions available for WGP filling and hydration-product growth. The performance of WGP-modified mortar is therefore governed by the interaction between WGP dosage and aggregate packing structure, yet this coupling has not been subjected to controlled multi-variable investigation.

Machine learning (ML) has emerged as a powerful surrogate modeling approach for predicting and optimizing properties of cementitious materials, where composition–property relationships are nonlinear and multi-variable. Javid et al. [14] demonstrated that ensemble tree models substantially outperformed conventional regression in predicting compressive strength of fiber-reinforced concrete under variable temperature conditions. Sun et al. [15] compared ML architectures for compressive strength prediction across diverse curing regimes and found that gradient-boosted decision trees consistently achieved higher generalization accuracy than empirical equations. Jin et al. [16] applied ML to high-performance concrete and highlighted the capacity of sequential boosting to capture higher-order interactions among mix variables without explicit feature engineering. Ababneh et al. [17] applied artificial neural networks to shear capacity prediction of recycled aggregate concrete, illustrating the broad applicability of ML across concrete types. Moein et al. [18] reviewed deep learning and classical ML applications in predicting concrete properties and identified physically constrained dataset construction as a key requirement for reliable extrapolation. Mungle et al. [19] further emphasized that surrogate models require experimentally bounded training domains and adequate dataset size to remain physically meaningful. While ML prediction of cementitious material strength is now well established, few studies have grounded data-driven optimization in experimentally verified microstructural mechanisms, and none has applied constrained XGBoost-PSO to jointly optimize WGP content and aggregate gradation.

The foregoing review identifies three principal research gaps. First, the coupled effect of WGP replacement ratio and fine-aggregate gradation on mortar performance has not been systematically quantified; most studies vary WGP dosage under a fixed aggregate gradation, obscuring the interaction between filling geometry and hydration kinetics. Second, existing ML models for WGP-modified materials are largely disconnected from microstructural evidence, limiting physical interpretation and confidence in optimization outcomes. Third, no study has combined mechanism-informed dataset construction with constrained PSO to generate physically feasible, strength-maximizing WGP–aggregate combinations. These gaps represent the challenge of moving from empirical replacement-ratio optimization toward coordinated, multi-scale mix design.

The present study addresses these gaps through four novel contributions: (i) a controlled multi-variable experimental matrix combining five fine-aggregate gradations with four WGP replacement levels to systematically quantify packing–WGP synergy; (ii) multi-scale SEM and MIP characterization of representative mixture subsets to establish mechanism-to-performance linkages; (iii) a rigorous comparison of four ML algorithms on a mechanism-bounded dataset, identifying XGBoost as the superior strength-prediction surrogate; and (iv) a constrained XGBoost-PSO framework that generates physically admissible, strength-maximizing WGP–aggregate combinations for subsequent experimental verification. The specific objectives are: (1) to quantify the synergistic effects of WGP dosage and aggregate packing on compressive and flexural strength; (2) to identify governing hydration, interfacial, and pore-structure mechanisms; (3) to compare representative ML regression models for multi-variable strength prediction; and (4) to demonstrate a mechanism-informed, constrained optimization route for sustainable mortar design.

## 2. Materials and Methods

### 2.1. Raw Materials

Ordinary Portland cement (P·O 42.5, Conch brand), conforming to the Chinese National Standard GB 175-2023 [20] for common Portland cement, was used as the primary binder. This grade is widely used in building construction and provides a representative matrix for assessing WGP incorporation.

**Figure 1:**
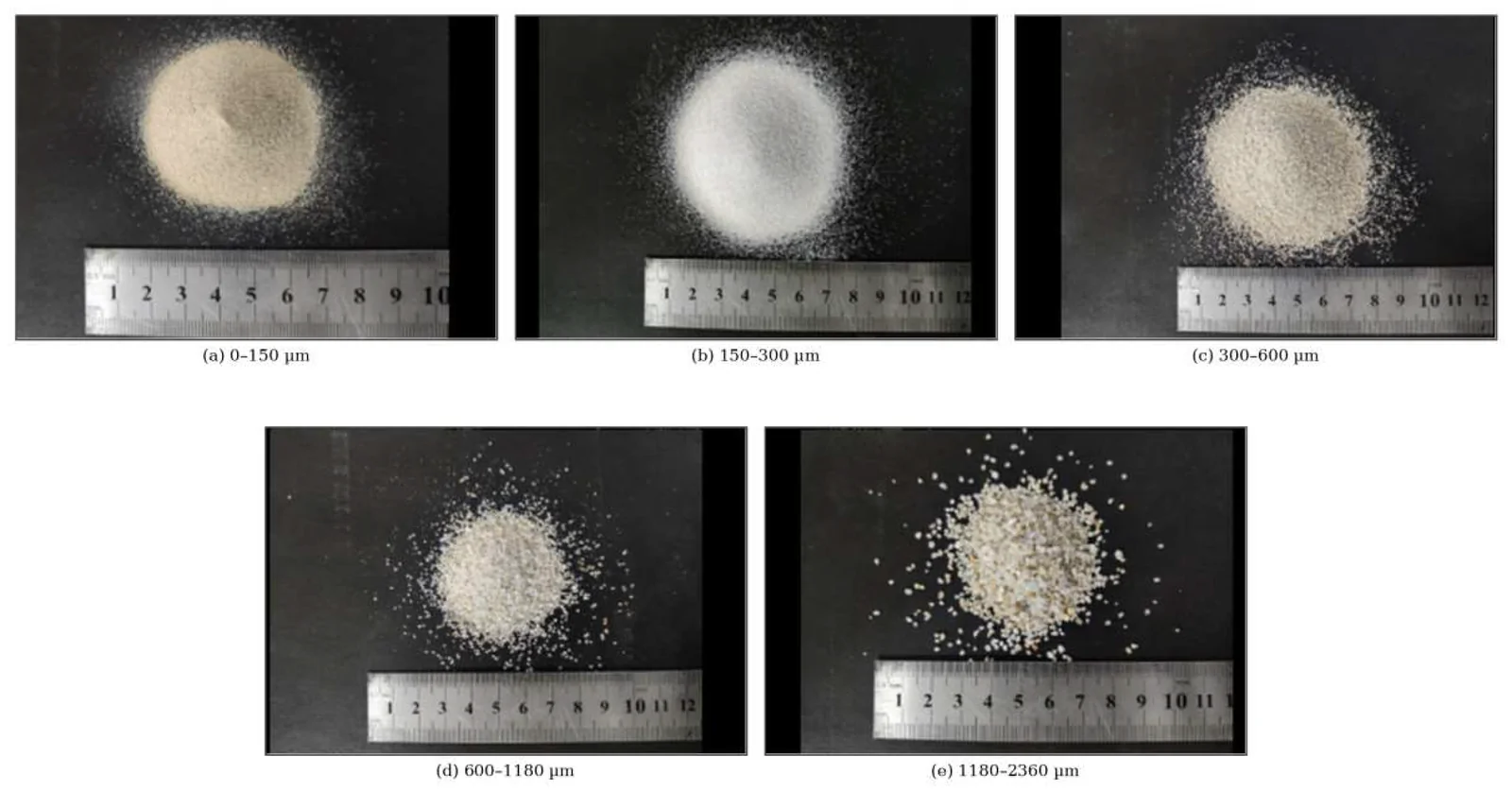
Five particle-size fractions of ISO standard sand obtained by sieve separation: (**a**) 0–150 µm; (**b**) 150–300 µm; (**c**) 300–600 µm; (**d**) 600–1180 µm; and (**e**) 1180–2360 µm. A steel ruler is shown for scale.

ISO standard sand (conforming to ISO 679:2009 [21] and the equivalent requirements of GB/T 17671-2021 [22]), supplied by Xiamen ISO Standard Sand Co., Ltd. (Xiamen, China), was used as the fine aggregate. The sand was separated by mechanical sieving into five fractions: 0–150 µm, 150–300 µm, 300–600 µm, 600–1180 µm, and 1180–2360 µm as shown in Figure 1. Using a single aggregate source minimized mineralogical variability while allowing systematic control of packing structure.

**Figure 2:**
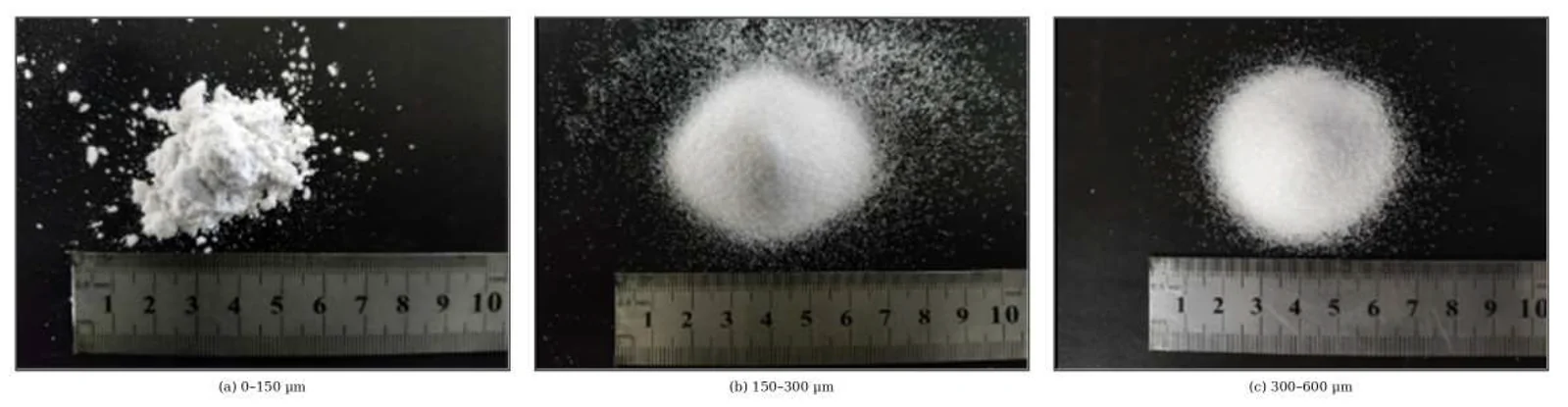
Waste glass powder fractions produced by crushing, ball milling, and dry sieving: (**a**) 0–150 µm; (**b**) 150–300 µm; and (**c**) 300–600 µm. A steel ruler is shown for scale.

WGP was sourced from post-consumer soda-lime glass (primarily discarded flat glass sheets and glass container cullet) collected from a glass recycling facility in Zhengzhou, Henan Province, China. The cullet was pre-cleaned to remove organic contamination and metallic inclusions, then crushed in a jaw crusher and further comminuted by ball milling. The resulting powder was dry-sieved into 0–150 µm, 150–300 µm, and 300–600 µm fractions as shown in Figure 2. Matching WGP and aggregate size intervals enabled equal-size-fraction substitution and preserved the intended gradation framework. In the mortar system, WGP functions primarily as a fine-aggregate replacement; where particle fineness and the alkaline environment are favorable, a secondary pozzolanic contribution through reaction of amorphous SiO_2_ with portlandite may additionally occur, leading to supplementary C-S-H formation and pore refinement. Tap water satisfying JGJ 63-2006 [23] was used for all mixtures.

The packing characteristics of the five aggregate gradations are quantified by the granulometric descriptors presented in Table 1(b). The fineness modulus (FM) increases monotonically from F (FM = 1.90) to C (FM = 3.10), confirming the intended progression from fine to coarse. Uniformity coefficients of M3 and C (Cu ≈ 4.7) indicate broader size distributions with greater potential for sequential particle nesting compared with F and M1 (Cu ≈ 3.1–3.3). The curvature coefficients indicate that M2 and C approach conventional well-graded thresholds (1 < Cc < 3), reflecting the intended controlled nature of the blended fractions. In the present study, packing behavior is characterized through these granulometric descriptors and corroborated by the observed mechanical and MIP responses. Direct measurement of loose and vibrated bulk packing density for each gradation is recommended as a complementary dataset for future research.

### 2.2. Mixture Design and Specimen Preparation

A constant water-to-cement ratio of 0.50 was used to isolate the effects of WGP dosage and aggregate gradation. Five gradations, denoted F, M1, M2, M3, and C in order of increasing coarseness (F = fine; M1, M2, M3 = medium, progressively coarser; C = coarse), were designed in accordance with GB/T 14684-2022 [24]. WGP replaced 0%, 8%, 10%, or 12% of the fine aggregate by mass. To preserve the particle-size framework of each series, WGP replaced the corresponding finest aggregate fractions rather than being added indiscriminately. Mixture proportions are given in Table 2, and mass fractions of the five gradations are given in Table 1(a). 

A total of 20 mixture combinations (5 gradations × 4 WGP levels) were prepared. Three replicate 40 mm × 40 mm × 160 mm prisms were cast for each mixture–age combination, yielding 60 prisms per testing age and 180 prisms in total across the three testing ages (7, 14, and 28 days). Mortars were mixed in a planetary mixer and cast into prisms in accordance with GB/T 17671-2021 [22]. Specimens were demolded after 24 h and cured at (20 ± 1) °C and relative humidity ≥ 95% until the respective testing age.

Flexural strength was determined using a three-point bending configuration at a loading rate of 50 N/s, applied at the mid-span of the prism, in accordance with GB/T 17671-2021 [22]. The two half-prisms resulting from each flexural test were subsequently used for compressive strength determination. A loading rate of 2400 N/s was applied through 40 mm × 40 mm steel platens, also in accordance with GB/T 17671-2021 [22]. All tests were conducted on a servo-controlled universal testing machine with a capacity of 300 kN, calibrated before each testing campaign. Three replicate values were recorded for each condition; results are reported as mean ± standard deviation, and error bars in all figures represent one standard deviation.

### 2.3. Microstructural Characterization

Representative fragments were collected from the inner regions of broken prisms after compressive testing to avoid boundary-affected zones. Hydration was arrested by immersion in anhydrous isopropanol for 72 h, followed by vacuum drying at 40 °C for 24 h to remove residual solvent without inducing thermal microcracking.

For SEM examination, relatively flat central fragments (maximum dimension ≤ 15 mm) were mounted on aluminum stubs with conductive carbon adhesive and sputter-coated with approximately 10 nm of gold to prevent charge accumulation. Imaging was performed under high vacuum at an accelerating voltage of 10–15 kV using a ZEISS Sigma 300 field-emission scanning electron microscope (Dongguan Sanben Precision Instrument Co., Ltd., Dongguan, China) equipped with an energy-dispersive X-ray spectroscopy (EDS) detector. SEM was used to assess hydration product morphology, particle contact quality, and interfacial transition zone (ITZ) characteristics for six representative 28-day mixtures: CS-F, CS-M3, CS-C, GCS12-F, GCS8-M3, and GCS10-C.

Pore structure was characterized by mercury intrusion porosimetry using a Micromeritics AutoPore V 9620 instrument (Dongguan Sanben Precision Instrument Co., Ltd., Dongguan, China). Fragments (maximum dimension ≤ 10 mm) were used to minimize macropore artefacts from sample boundaries. Prior to testing, specimens were vacuum dried at 60 °C for 24 h to remove free moisture. Intrusion pressure was increased stepwise from approximately 0.003 MPa to 413 MPa, corresponding to pore entry diameters from approximately 360 µm to 3 nm based on the Washburn equation, using a mercury contact angle of 130° and surface tension of 485 mN/m. Total porosity, volume-based median pore diameter, area-based median pore diameter, and mean pore diameter (4V/A) were extracted from the intrusion curves and are reported in Table 3.

Statistical analysis. All mechanical test results are reported as mean ± standard deviation of three replicate specimens. One-way analysis of variance (ANOVA) was applied at a significance level of α = 0.05 to test for statistically significant differences in 28-day strength among gradation series within each WGP level. Where ANOVA indicated significant differences, Tukey’s honestly significant difference (HSD) post hoc test was used for pairwise comparisons. ML model performance was evaluated using the coefficient of determination (R^2^), root-mean-square error (RMSE), and mean absolute error (MAE) on the held-out 20% test subset.

### 2.4. Data-Driven Prediction and Optimization Framework

#### 2.4.1. Regression Models

Four regression algorithms were compared under an identical data partition: linear regression (LR), support vector regression (SVR), random forest (RF), and extreme gradient boosting (XGBoost). LR served as an interpretable linear baseline. SVR represented nonlinear kernel-based regression, while RF and XGBoost represented tree-ensemble approaches capable of capturing interactions among WGP content, aggregate gradation, and curing age.

RF reduces variance by averaging multiple independently constructed decision trees and is robust to multicollinearity among mixture variables. SVR maps the input variables into a higher-dimensional feature space and identifies a regularized regression function within a prescribed error margin. XGBoost builds trees sequentially to correct residual errors and includes regularization to control model complexity. This structure is well suited to tabular datasets with nonlinear feature coupling and was evaluated as a potential optimization surrogate.

#### 2.4.2. Dataset Construction and Validation

Seven input variables were used: WGP replacement ratio, the mass fractions of the five aggregate size intervals, and curing age. The five aggregate fractions were constrained to sum to 100%. Compressive strength was the primary prediction target; flexural strength was modeled separately for use in the constrained optimization objective.

The experimental program produced 60 mixture–age observations (20 mixture combinations × 3 testing ages). These 60 data points span the complete experimental design space but represent a relatively sparse sampling for direct ML training given the seven-dimensional input space and the nonlinear interactions among aggregate fractions. To improve within-domain continuity and reduce model variance caused by the limited dataset, 500 supplementary interpolation samples were generated by a mathematical procedure calibrated to the experimental observations. Specifically, a smooth response surface was fitted to the 60 experimental observations using locally weighted quadratic regression within the bounded compositional space. New sample points were then drawn at systematically distributed locations within the convex hull of the experimental design, and their strength values were computed from the fitted surface and perturbed by Gaussian noise with standard deviation equal to the average within-replicate measurement variability (compressive: ±1.1 MPa; flexural: ±0.3 MPa) to preserve the statistical character of the experimental scatter. These supplementary points are located strictly within the experimental design domain; no extrapolation beyond the tested WGP range (0–12%) or aggregate fraction bounds was performed during augmentation. These samples are not independent experimental observations and do not constitute additional physical evidence; their purpose is solely to provide a denser representation of the known experimental trends within the validated domain, improving the numerical stability of tree-ensemble training.

To assess the sensitivity of model performance to the augmentation procedure, XGBoost was trained and evaluated under two conditions: (i) on the 60 experimental points alone with five-fold cross-validation and (ii) on the combined 560-point dataset (60 experimental + 500 augmented) with an 80/20 split and five-fold cross-validation of the training subset. Under condition (i), XGBoost achieved R^2^ = 0.906, RMSE = 1.23 MPa, and MAE = 0.89 MPa on the held-out experimental fold. Under condition (ii), XGBoost achieved R^2^ = 0.9346, RMSE = 0.9860 MPa, and MAE = 0.6943 MPa on the 20% held-out subset. The consistent ranking of models and directional agreement between the two conditions confirm that the augmentation procedure improved numerical smoothness without introducing systematic bias into the comparative evaluation. Nevertheless, the marginal improvement in absolute metrics should be interpreted with caution: the augmented dataset remains a mathematical elaboration of 60 experimental measurements, and the resulting models should be regarded as engineering surrogates for interpolation within the defined material domain. Extrapolation beyond the experimental composition space requires independent experimental verification (Section 3.4).

The combined dataset was split into 80% training and 20% testing subsets before model tuning. Five-fold cross-validation was applied to the training subset only; the 20% test subset was withheld until final model evaluation to provide an unbiased performance estimate. Performance was evaluated using R^2^, RMSE, and MAE.

#### 2.4.3. XGBoost-PSO Mixture Optimization

Because XGBoost achieved the best prediction accuracy, it was coupled with particle swarm optimization (PSO). PSO generated candidate WGP–aggregate combinations, XGBoost predicted their compressive and flexural strengths, and infeasible candidates were penalized. The design variables were WGP replacement ratio, five aggregate-size fractions, and curing age. The principal objective was to maximize 28-day compressive strength while retaining adequate flexural performance and a physically admissible continuous gradation.

The optimization objective was defined as follows:

(1) f(x) = f_comp + α · f_comp^2^

where f(x) is the objective value, f_comp is the XGBoost-predicted 28-day compressive strength, and α is a quadratic weighting coefficient used to increase discrimination in the high-strength region. Soft penalties were applied to infeasible solutions.

The following constraints were imposed to preserve physical and engineering feasibility.

(i) Aggregate gradation: the five aggregate fractions were required to sum to 100%, and each fraction was maintained at not less than 2% to avoid degenerate grading.

(2) s_1_ + s_2_ + s_3_ + s_4_ + s_5_ = 100%

(3) min(s_1_, s_2_, s_3_, s_4_, s_5_) ≥ 2%

where s_1_–s_5_ are the mass fractions of the 0–150 µm, 150–300 µm, 300–600 µm, 600–1180 µm, and 1180–2360 µm fractions, respectively.

(ii) WGP replacement ratio:

(4) 5% ≤ glass ≤ 30%

(iii) Flexural strength:

(5) f_flex ≥ 5.0 MPa

(iv) Curing age: candidate ages were restricted to 7, 14, or 28 days.

The swarm contained 120 particles and was run for a maximum of 300 iterations. Table 4 summarizes the search ranges. The WGP bounds were chosen to exclude negligible modification at very low dosage and excessive dilution at high dosage; together with the size-fraction bounds of 2–90%, they define a search space wider than the tested domain (WGP 0–12%, size fractions as in Table 1(a)). Because PSO is stochastic and the surrogate is calibrated only within the tested domain, a candidate located outside that domain is treated as a hypothesis requiring experimental confirmation (Section 3.4).

## 3. Results and Discussion

### 3.1. Mechanical Performance Governed by WGP Dosage and Aggregate Packing

#### 3.1.1. Compressive Strength

**Figure 3:**
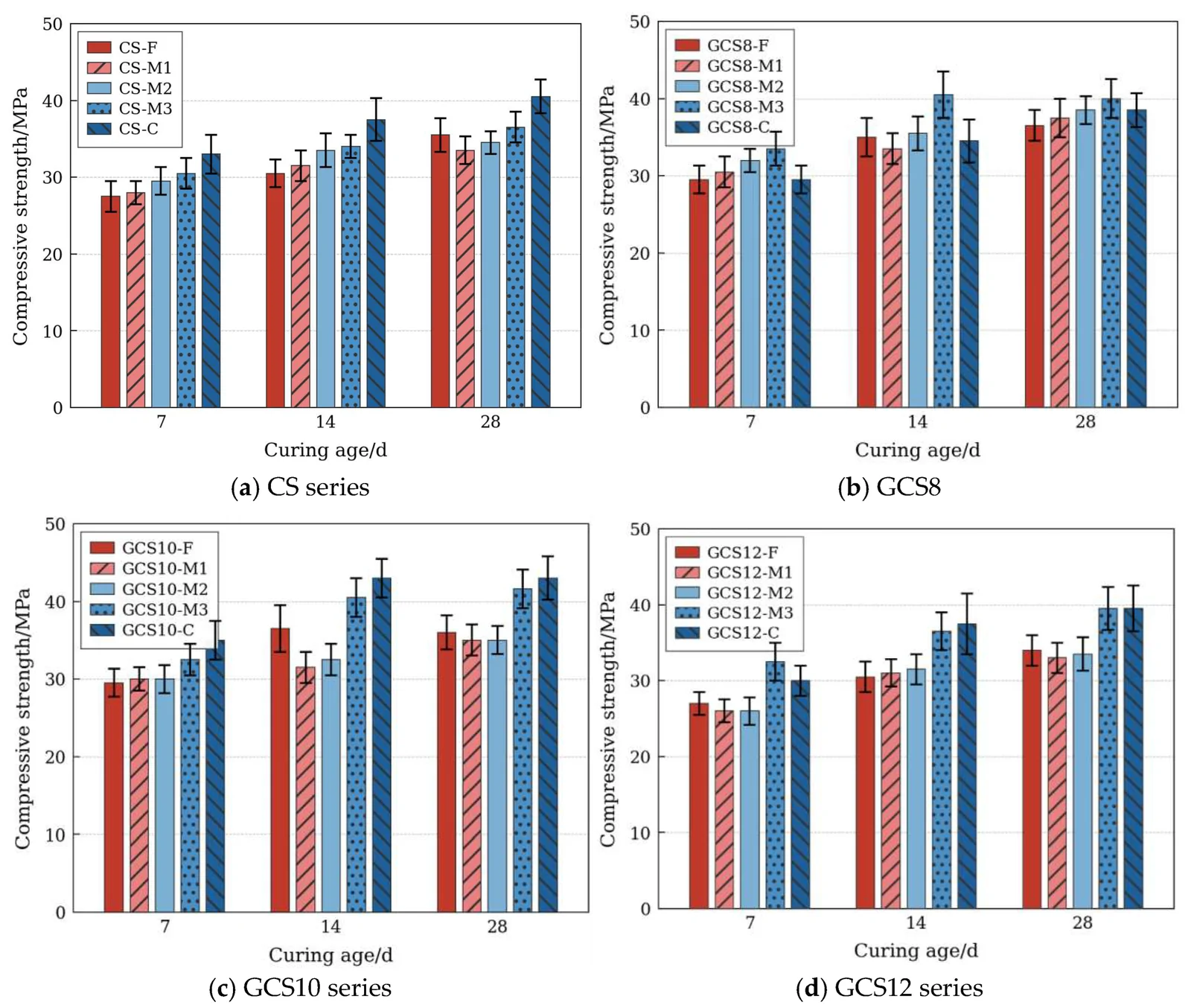
Compressive strengths at 7, 14, and 28 days for WGP-modified cement mortars across the five aggregate gradations (F, M1, M2, M3, C): (**a**) CS series, 0% WGP (reference); (**b**) GCS8 series, 8% WGP; (**c**) GCS10 series, 10% WGP; (**d**) GCS12 series, 12% WGP. Error bars represent one standard deviation of three replicate specimens. Within each panel, gradation labels follow the order F, M1, M2, M3, C, from finest to coarsest.

As shown in Figure 3, compressive strength increased with curing age for every mixture because of continued cement hydration and development of a load-bearing C-S-H network. The magnitude of strength gain, however, depended on both WGP dosage and aggregate gradation, confirming that these parameters interacted rather than acted independently. GCS10-M3 achieved the highest 28-day compressive strength of 41.6 MPa, 14.0% above CS-M3. The M3 skeleton provided sufficient interstitial volume for WGP particles to fill voids and participate in secondary reaction, thereby improving particle packing, pore refinement, and apparent matrix continuity. SEM observations (Section 3.2.1) show fewer connected macropores and improved apparent particle contacts in WGP-containing specimens, consistent with micro-filling and secondary hydration contributions to a less porous transition zone. Definitive quantification of ITZ thickness and direct chemical confirmation of secondary C-S-H distribution within this zone would require EDS line scanning or selective paste extraction and constitute a priority for future investigation.

The beneficial effect was not monotonic. At 12% replacement, compressive strength generally decreased because excessive fine-particle addition disrupted the packing balance and increased the unreacted WGP fraction, outweighing the additional filling and pozzolanic contributions. Aggregate gradation also controlled the response: intermediate and coarse-leaning gradations generally used WGP more effectively than an excessively fine skeleton, which limited particle rearrangement and hydration-product growth. The optimum WGP dosage should therefore be selected together with aggregate packing rather than treated as a fixed material constant.

The 14.0% compressive strength gain achieved by GCS10-M3 is consistent with improvements reported for WGP substitution in the literature. Ibrahim [7] reported up to 20% compressive strength improvement at 15% WGP cement replacement in ternary blends containing silica fume and fly ash, where combined SCM effect amplifies pozzolanic densification. Li et al. [12] observed that WGP dosages up to 10% improved pervious concrete compressive strength, with declines at higher dosages attributed to matrix dilution—a trend that mirrors the present findings at 12% aggregate replacement. The present results extend this pattern to aggregate-substitution mode, confirming that the direction of WGP effect is maintained regardless of substitution strategy, while the optimum dosage is modulated by the aggregate gradation environment. The strong performance of the M3 skeleton is attributable to its intermediate coarseness (FM = 2.50, Cu = 4.7), which provides sufficient interstitial volume for WGP particle accommodation without excessive paste demand, consistent with the role of well-graded aggregate in maximizing packing density described by Jani and Hogland [10].

#### 3.1.2. Flexural Strength

**Figure 4:**
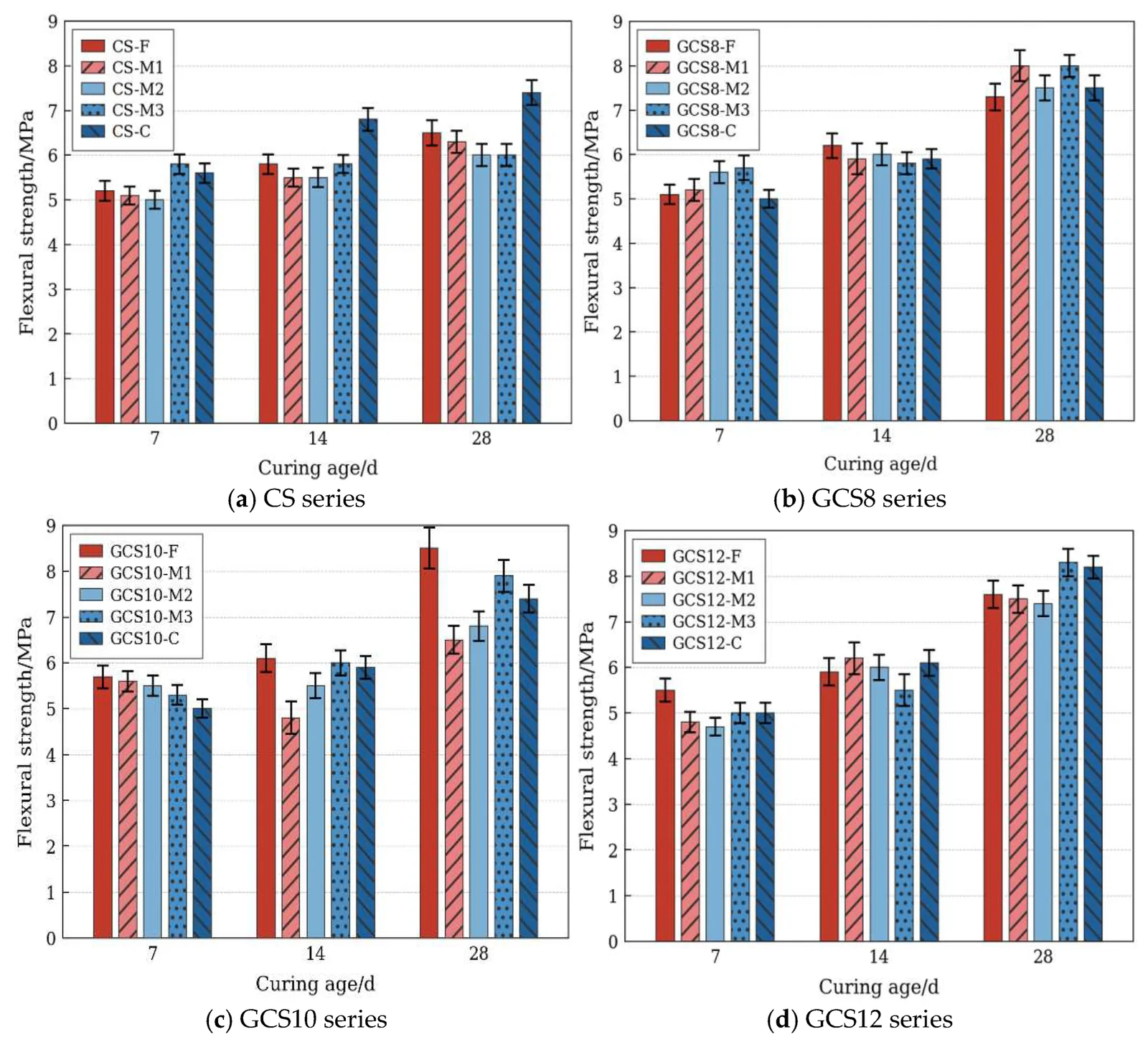
Flexural strengths at 7, 14, and 28 days for WGP-modified cement mortars across the five aggregate gradations (F, M1, M2, M3, C): (**a**) CS series, 0% WGP (reference); (**b**) GCS8 series, 8% WGP; (**c**) GCS10 series, 10% WGP; (**d**) GCS12 series, 12% WGP. Error bars represent one standard deviation of three replicate specimens.

Figure 4 shows that flexural strength was more sensitive to WGP incorporation than compressive strength. At 28 days, all WGP-containing mixtures exceeded their corresponding references, with a maximum increase of 42.9%. This stronger relative response reflects the governing failure mechanism: flexural strength is highly sensitive to microdefects and bond quality in the ITZ, whereas compressive resistance is controlled more broadly by the bulk matrix.

Fine WGP reduced local stress concentrations by filling ITZ voids, while secondary C-S-H generation strengthened the aggregate–paste interface and bridged microdefects. These mechanisms delayed crack initiation and propagation under bending. The M3 gradation provided effective aggregate interlocking and a favorable spatial environment for WGP filling, whereas excessive WGP or overly fine gradation reduced the benefit through incomplete hydration and less efficient stress transfer.

The proportionally larger flexural strength gain (42.9%) compared with the compressive gain (14.0%) is consistent with observations by Mohammed and Hama [8], who reported that WGP incorporation improved flexural strength more substantially than compressive strength in green concrete systems. This differential response reflects the greater sensitivity of flexural failure to microdefect density and bond quality at the ITZ. Balasubramanian et al. [6] similarly attributed disproportionate flexural improvement to fine-particle filling of ITZ voids, a mechanism supported by the present SEM analysis. The M3 gradation amplified this effect through favorable aggregate interlocking geometry; this observation has direct implications for mix design: gradation selection should be considered alongside WGP dosage when flexural performance is a governing criterion.

### 3.2. Microstructural Mechanisms

#### 3.2.1. SEM Observations

Six representative 28-day mixtures (CS-F, CS-M3, CS-C, GCS12-F, GCS8-M3, and GCS10-C) were examined via SEM. The micrographs reveal qualitative differences in matrix density, hydration product morphology, and apparent particle contact quality that are consistent with the observed mechanical trends, though the following discussion distinguishes between features directly visible in the micrographs and inferences based on established hydration chemistry.

**Figure 5:**
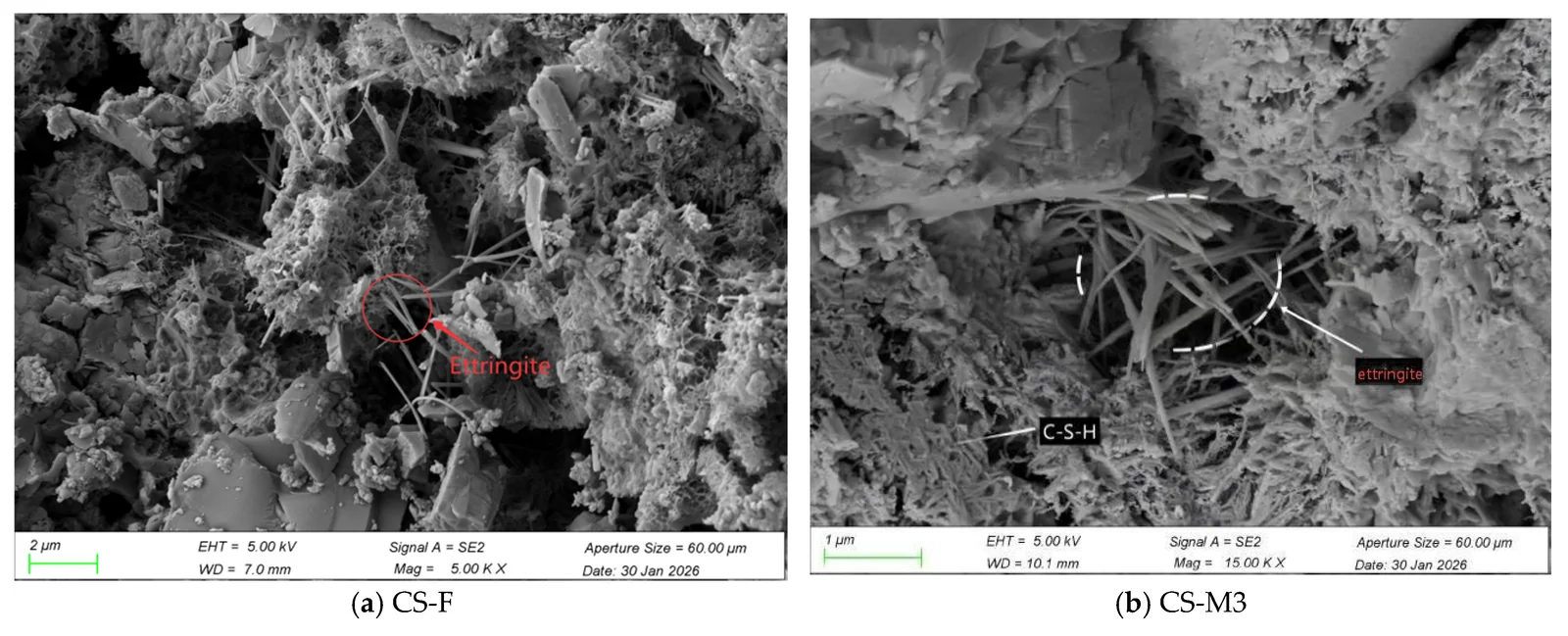

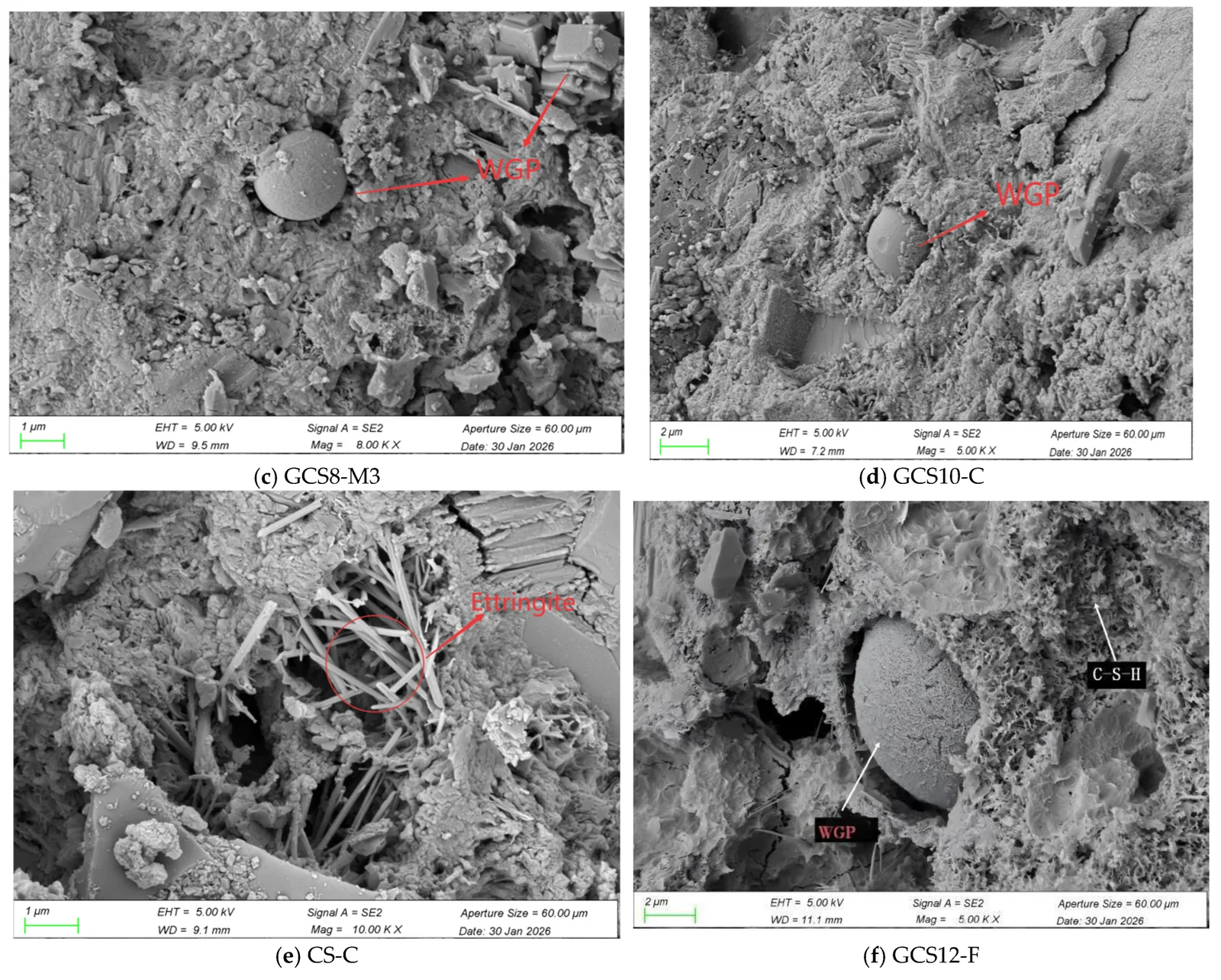
SEM micrographs of selected 28-day mortars: (**a**) CS-F—reference mortar with fine gradation, showing inter-particle voids (V) and loosely packed hydration products (H); (**b**) CS-M3—reference mortar with M3 gradation, showing improved particle contact (PC) and denser hydration product network; (**c**) GCS8-M3—8% WGP with M3 gradation, showing close aggregate contacts and a continuous low-porosity matrix with WGP filling sites (WF); (**d**) GCS10-C—10% WGP with coarse gradation, showing WGP particles at inter-aggregate positions within a dense matrix; (**e**) CS-C—reference mortar with coarse gradation, illustrating naturally compact structure; (**f**) GCS12-F—12% WGP with fine gradation, showing locally unreacted WGP particles (UPs) within a comparatively porous matrix. Scale bars are included in each micrograph. Key microstructural features are indicated by arrows or dashed-box markers in the original micrographs.

In CS-F (Figure 5a), the matrix displays a relatively porous network with irregular void spaces (V) between hydration products, and particle contacts appear less intimate, consistent with the dominantly fine gradation providing limited mechanical interlocking. CS-M3 (Figure 5b) shows a noticeably denser arrangement, with hydration products (H) occupying a greater proportion of the visible interstitial space; aggregate particles appear more closely nested, consistent with the intermediate gradation (FM = 2.50) providing better sequential filling of void space.

GCS8-M3 (Figure 5c) exhibits the densest matrix among the examined specimens. Aggregate particles are closely contacted (PC), and the surrounding hydration product zone shows reduced visible porosity relative to CS-M3. Fine particles—morphologically consistent with partially or fully reacted WGP—are observed occupying spaces between aggregate and cement hydration products at WGP filling sites (WF). Whether these particles fully participated in pozzolanic reaction cannot be determined from SEM morphology alone; EDS or TEM analysis would be required to distinguish reacted glass-derived C-S-H from unreacted glass or primary cement hydration products. Nevertheless, the reduced connected void space and improved apparent matrix continuity are consistent with combined micro-filling and secondary hydration contributions.

GCS10-C (Figure 5d) similarly shows a dense matrix, with WGP particles situated at aggregate contacts and in inter-aggregate voids. The coarse C gradation provides larger interstitial void spaces than the F gradation, which appears to accommodate WGP particles without disrupting the coarser aggregate skeleton.

CS-C (Figure 5e) displays a comparatively compact reference matrix, attributable to the inherently lower paste demand of the coarse gradation. GCS12-F (Figure 5f) shows locally unreacted or partially reacted WGP particles (UPs) within a comparatively porous matrix. At 12% replacement in the fine F gradation, excess fine particles appear to occupy space without complete integration into the hydration product network, consistent with the mechanical decline observed at this dosage.

Overall, the SEM observations support the interpretation that moderate WGP incorporation in intermediate-to-coarse gradations improves apparent matrix density and particle contact quality. However, the absence of local EDS chemical mapping, portlandite distribution analysis, and ITZ thickness measurement means that conclusions regarding specific chemical mechanisms (pozzolanic reaction extent, secondary C-S-H formation, portlandite depletion at the glass–paste interface) remain inferences supported by the broader hydration chemistry literature rather than direct experimental evidence from this study.

#### 3.2.2. Pore Structure Characterized by MIP

**Figure 6:**
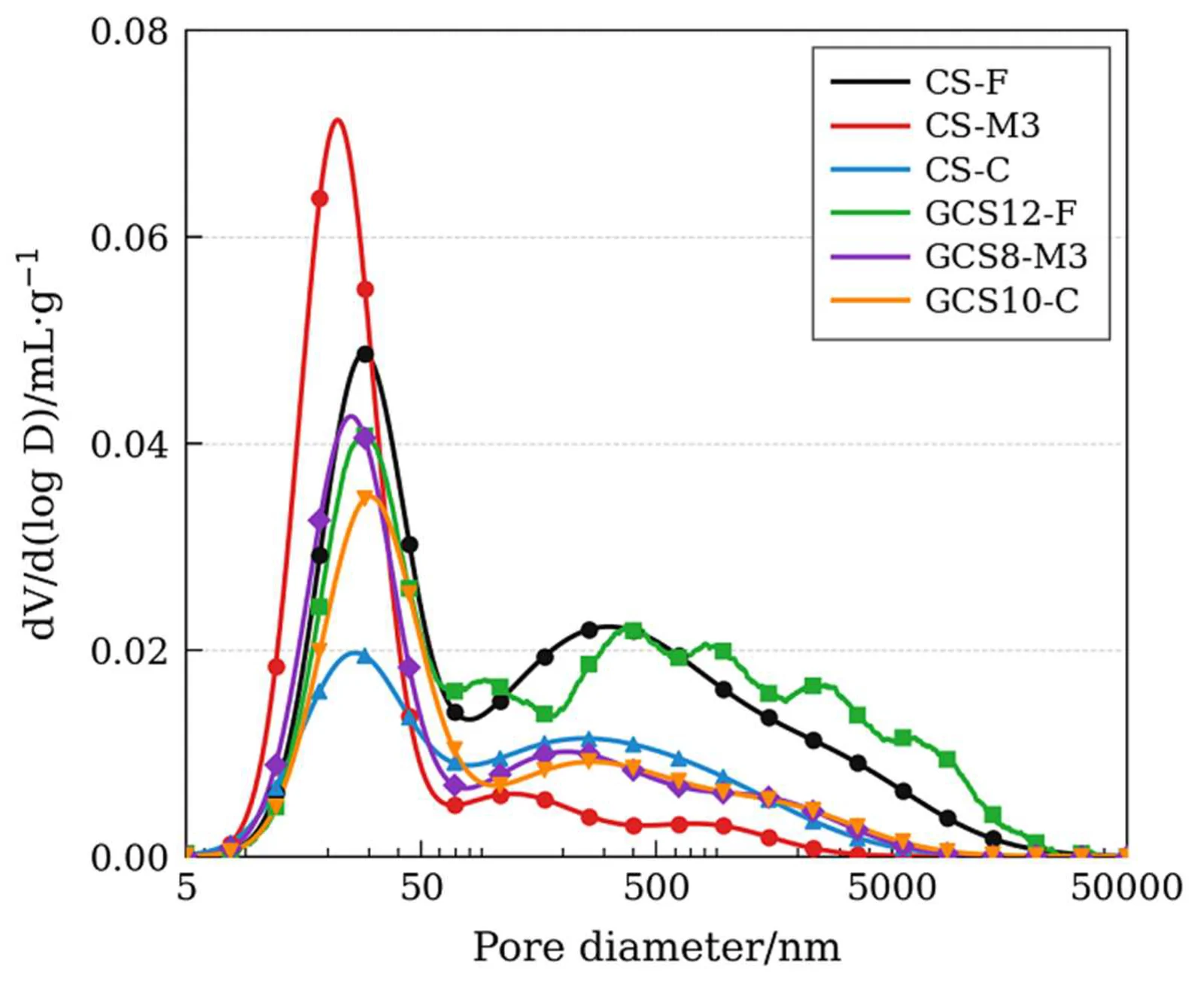
Differential mercury intrusion pore-size distributions plotted against pore entry diameter (logarithmic scale) for six selected 28-day mortars: CS-F (fine reference), CS-M3 (medium-coarse reference), CS-C (coarse reference), GCS12-F (12% WGP, fine gradation—highest porosity case), GCS8-M3 (8% WGP, M3 gradation—optimal case), and GCS10-C (10% WGP, coarse gradation). Dominant intrusion peaks in WGP-containing mixtures with favorable gradation (GCS8-M3, GCS10-C) shift toward smaller pore diameters and decrease in height relative to their respective references, indicating pore refinement. GCS12-F deviates from this trend, with a peak shift toward larger pore sizes, consistent with disrupted packing and incomplete hydration. Quantitative pore parameters are given in Table 3.

MIP was performed on the same six mixtures. Figure 6 and Table 3 show marked differences in pore-size distribution and total porosity. The reference mortars had dominant pore populations around 50–100 nm. CS-F also contained a secondary macropore population above 500 nm, while CS-C showed a lower, finer principal peak, indicating a denser structure.

After WGP incorporation, the main intrusion peaks generally shifted toward smaller pore sizes and decreased in intensity, indicating transformation of larger harmful pores into finer pores. This redistribution is mechanically important because large connected pores act as preferential sites for stress concentration and crack initiation. GCS8-M3 combined lower porosity with a reduced mean pore diameter, whereas GCS12-F showed increased porosity and larger characteristic pores because excessive WGP and the fine skeleton restricted hydration-product development.

The porosity sequence of the reference mortars was CS-F > CS-M3 > CS-C. GCS8-M3 reduced porosity by 2.0 percentage points relative to CS-M3. Although GCS10-C had slightly higher porosity than CS-C, its absolute porosity remained low. These results demonstrate that pore refinement depends on the balance between aggregate packing, filling, and secondary hydration rather than on WGP dosage alone.

Mechanistic interpretation of pore structure evolution. The observed shift of the dominant intrusion peak toward smaller pore diameters in WGP-containing mixtures is attributable to two concurrent processes. The first is physical micro-filling: WGP particles within the 0–300 µm size range occupy macropores and large mesopores within the aggregate skeleton, reducing the population of pores accessible at low intrusion pressures. The second process is chemical pore refinement through secondary C-S-H formation: amorphous SiO_2_ from WGP reacts with Ca(OH)_2_ generated during cement hydration in the alkaline pore solution, yielding additional C-S-H gel that deposits within pre-existing pores and at the glass–paste interface. This reaction is well documented for sufficiently fine glass particles (D < 150 µm) in cement matrices [10] and is consistent with the pore-size redistribution observed here. However, the relative contributions of physical filling and pozzolanic densification cannot be separated directly from MIP data alone; complementary XRD or thermogravimetric analysis (TGA) of portlandite content as a function of WGP dosage and curing age would be required to quantify the chemical component.

The divergent behavior of GCS12-F is mechanistically significant. The simultaneous increase in total porosity (13.7% versus 12.8% for CS-F) and shift of the median pore diameter toward larger values (137.3 nm versus 74.0 nm) indicates that the fine F gradation combined with 12% WGP exceeds the threshold at which physical filling can compensate for cement content dilution and the spatial restriction imposed on hydration product growth. This mechanism explains why gradation selection is not merely an aesthetic choice but a structural constraint on WGP effectiveness, and it provides the physical rationale for the experimentally observed optimum at M3 rather than F.

**Table 4:** Search ranges used in the XGBoost-PSO optimization. Symbols: glass = WGP replacement ratio by mass relative to total fine aggregate; s_1_–s_5_ = mass fractions of the 0–150, 150–300, 300–600, 600–1180, and 1180–2360 µm aggregate size fractions, respectively, constrained to sum to 100%; age = curing age in days.

Parameter	Symbol	Minimum Value	Maximum
WGP replacement ratio (%)	glass	5	30
Fine aggregate 0–150 µm (%)	s_1_	2	90
Fine aggregate 150–300 µm (%)	s_2_	2	90
Fine aggregate 300–600 µm (%)	s_3_	2	90
Fine aggregate 600–1180 µm (%)	s_4_	2	90
Fine aggregate 1180–2360 µm (%)	s_5_	2	90
Curing age (d)	age	7	28

#### 3.2.3. Multiscale Structure–Property Relationship

The mechanical, SEM, and MIP results form a consistent, multiscale mechanistic picture, though it should be noted that the SEM evidence is qualitative and the chemical components of the proposed mechanisms require further analytical confirmation.

At the particle scale, aggregate gradation defines the initial packing geometry. The FM and Cu values in Table 1(b) indicate that M3 and C gradations achieve broader particle-size distributions, providing more extensive sequential nesting opportunities for WGP particles of corresponding size. The larger interstitial voids in M3 and C accommodate WGP particles without disrupting the load-bearing aggregate skeleton, whereas the finer F gradation offers limited void space for independent WGP accommodation.

At the hydration scale, moderate WGP—where particle size is within the reactive range and the local Ca(OH)_2_ supply is adequate—contributes secondary C-S-H through pozzolanic reaction, as supported by pore-size redistribution in MIP data and by qualitative SEM evidence of reduced connected voids. The extent of this reaction is expected to increase with decreasing WGP particle size and increasing curing age, consistent with the observed age-dependent strength gain differential between WGP-containing and reference mortars.

At the pore scale, the combination of physical filling and secondary C-S-H deposition refines the pore network: total porosity decreases and the dominant pore population shifts toward smaller, less hydraulically connected pores (Table 3). Large capillary pores (>100 nm), which act as preferential crack initiation sites under mechanical loading, are partially converted to finer pores, reducing stress concentration.

At the interface scale, the improvement in apparent particle contact quality visible under SEM—most pronounced in GCS8-M3—suggests reduced local porosity in the aggregate–paste transition zone, consistent with improved stress transfer efficiency. The disproportionate flexural strength gain relative to compressive strength across WGP-modified mixtures is mechanistically consistent with this ITZ effect, since flexural failure is more sensitive to bond quality and microdefect density at the aggregate–paste interface than is compressive failure, which is governed more broadly by bulk matrix porosity.

Excessive WGP (12%) reverses these benefits in fine gradations by disrupting the packing balance, reducing cement content below the threshold required for adequate hydration product connectivity and filling pore space with unreacted glass particles that contribute neither strength nor pozzolanic product at 28 days. The optimal performance is therefore produced not by a fixed WGP dosage, but by a coordinated combination of WGP content and aggregate gradation that balances void accommodation, hydration product growth, and cement dilution.

### 3.3. Machine-Learning Prediction Performance

#### Compressive Strength Prediction

**Figure 7:**
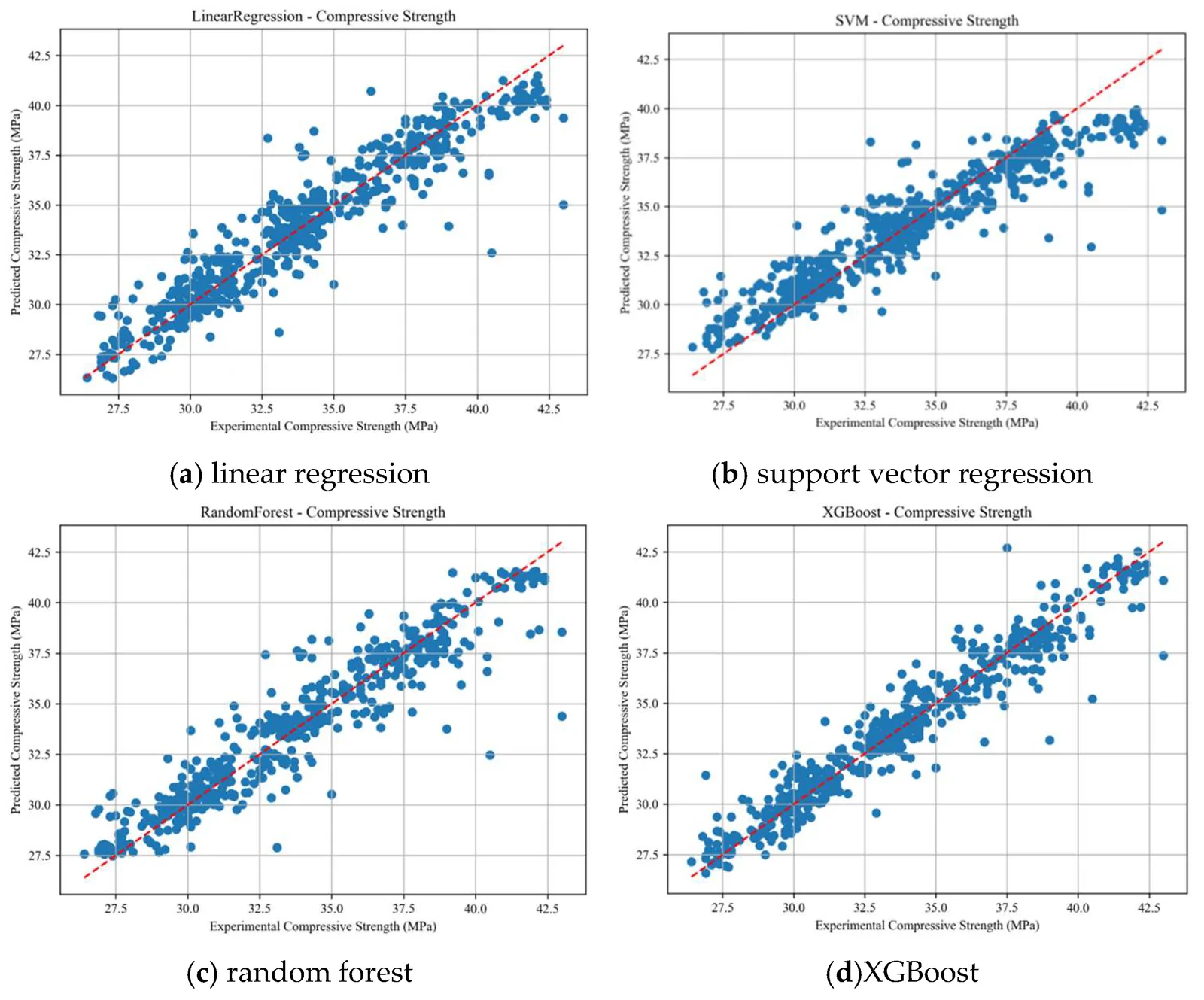
Predicted versus measured compressive strength (MPa) for the held-out 20% test subset: (**a**) linear regression (LR); (**b**) support vector regression (SVR); (**c**) random forest (RF); (**d**) XGBoost. The dashed line represents perfect prediction (slope = 1, intercept = 0); R^2^, RMSE, and MAE values are shown in each panel. Data points are colored by curing age (7, 14, and 28 days).

As shown in Figure 7, all four models achieved R^2^ values above 0.85, but XGBoost provided the best overall accuracy (R^2^ = 0.9346, RMSE = 0.9860 MPa, and MAE = 0.6943 MPa). RF, LR, and SVR yielded R^2^ values of 0.8896, 0.8845, and 0.8612, respectively. The superior performance of XGBoost indicates that tree boosting captured nonlinear interactions among WGP dosage, aggregate fractions, and curing age more effectively than the linear and kernel-based alternatives. Nevertheless, the model is an interpolation-oriented engineering surrogate whose applicability is bounded by the experimental and augmented design domain.

The superiority of XGBoost over LR, SVR, and RF is consistent with the broader ML literature on cementitious materials. Sun et al. [15] found that gradient-boosted decision trees outperformed polynomial regression and artificial neural networks across varied curing conditions, and Jin et al. [16] attributed the advantage of sequential boosting to its capacity to capture higher-order mixture–variable interactions without explicit feature engineering. The R^2^ of 0.9346 obtained here is comparable to values reported by Javid et al. [14] (R^2^ ≈ 0.93–0.97 for XGBoost applied to fiber-reinforced concrete) and Mungle et al. [19] (R^2^ ≈ 0.91–0.95 under variable curing), confirming that model performance is consistent with published benchmarks for datasets of similar size and compositional complexity. The relatively modest advantage of XGBoost over RF (ΔR^2^ ≈ 0.045) suggests that both ensemble approaches adequately capture aggregate gradation–WGP interaction, but sequential error correction in XGBoost provides additional precision in the mid-strength range where packing–hydration coupling is most complex.

### 3.4. XGBoost-PSO Mixture Optimization

The optimization converged within 300 iterations to a candidate mixture containing 14.86% WGP and a fines-rich bimodal gradation (Table 5). The predicted 28-day compressive and flexural strengths were 41.10 MPa and 8.23 MPa, respectively. This WGP content exceeds both the best experimental level (10%) and the highest tested level (12%), because PSO searched a continuous domain whose WGP bound (30%) lies outside the experimental range.

The result also indicates that the optimum WGP content depends on the accompanying aggregate skeleton. Larger 600–1180 µm and 1180–2360 µm fractions would provide interstitial volume that partially offsets the spatial crowding associated with a higher WGP dosage. The optimization is therefore consistent with the experimentally observed packing–WGP synergy.

Position of the candidate relative to the tested domain. The candidate lies outside the experimentally verified domain in three respects. Its WGP content (14.86%) exceeds the highest tested level (12%), its 0–150 µm fraction (21.98%) is more than twice the largest tested value (10%), and its 300–600 µm fraction (9.25%) is below the smallest tested value (15%), while its 600–1180 µm fraction (30.29%) sits at the upper edge of the tested range. The fineness modulus of the candidate, calculated as in Table 1(b), is 2.17, between those of M1 (2.15) and M2 (2.25). Therefore, its distinguishing feature is not coarseness but a bimodal profile in which a 600–2360 µm skeleton (53.42% in total) is combined with a large 0–150 µm fraction and a depleted 300–600 µm band. Because XGBoost is an ensemble of regression trees, its prediction stops changing once an input moves beyond the outermost split threshold learned from the training data. For WGP contents above 12% and for size fractions outside the tested bounds, the surrogate therefore returns the response found at the corresponding edge of the training domain, and the exact coordinates of the optimum in this region are not determined by the model.

The predicted compressive strength of the candidate (41.10 MPa) is 0.5 MPa below the measured strength of GCS10-M3 (41.6 MPa), a difference smaller than both the model RMSE (0.99 MPa) and the average within-replicate scatter (1.1 MPa). Therefore, the candidate is best regarded as a testable hypothesis, namely that a fines-rich bimodal skeleton can accommodate about 15% WGP with no strength loss relative to the best tested mixture, rather than as a mixture expected to outperform it. With the batch composition of Table 2, 14.86% WGP corresponds to 178 g of glass per 1200 g of aggregate, about 1.5 times the 120 g used in GCS10-M3, and under the substitution rule of Section 2.2 the entire glass dose would fall within the 0–150 µm fraction (264 g in total).

Experimental validation program. The confirmatory program, which is in progress and will be reported in a subsequent study, uses the materials, water-to-cement ratio (0.50), mixing, curing, and strength tests described in Section 2 [22]. Four mixtures are included. The first is the PSO candidate, batched from sieved sand fractions and WGP in the proportions of Table 5. The second keeps the candidate gradation but limits WGP to 12%, the upper bound of the training data, so that its comparison with the first isolates the effect of raising WGP beyond the tested range, which the surrogate cannot resolve. The third and fourth are GCS12-M3 and GCS10-M3, recast in the same campaign, so that the new gradation is compared with the best tested gradation at equal WGP content and with the best tested mixture without batch-to-batch variability. Compressive and flexural strengths are measured on three prisms per mixture at 7, 14, and 28 days, and differences are assessed with the ANOVA and Tukey procedure of Section 2.3. The prediction is regarded as confirmed if the mean 28-day compressive strength of the candidate lies within ±2 RMSE (±1.97 MPa) of the predicted value, that is, 39.1–43.1 MPa, and its flexural strength satisfies Equation (5). Because the candidate contains more than twice the 0–150 µm content of any tested gradation at a fixed water-to-cement ratio, and workability was not measured in the present study, mortar flow is determined following GB/T 2419-2005 [25]. MIP and SEM on the 28-day candidate, performed as in Section 2.3, will show whether the pore refinement identified in Section 3.2.2 persists at the higher WGP content. The new results will then be added to the training data, and the optimization will be repeated with the search bounds of Table 4, restricted to the enlarged experimental domain.

Implications for practical application. If confirmed, the candidate would raise the recycled-glass content by about half relative to GCS10-M3, at a comparable strength level. Because WGP replaces natural sand rather than cement, the resulting benefit lies in sand conservation and the diversion of waste glass from landfill, whereas the cement content remains 400 g per batch and the cement-related CO_2_ burden is unchanged. Three conditions govern transfer to production. First, the candidate gradation must be reproducible at scale. Dry-mixed mortar plants commonly dry and screen sand into several size fractions that are stored and weigh-batched separately, and in the candidate the 0–150 µm fraction would be supplied largely by WGP, so the glass can be handled as a designated fines component; the additional milling energy needed to bring all of the glass below 150 µm belongs in the cost–benefit analysis noted in Section 3.5. Second, alkali–silica reaction (ASR) must be excluded. The ASR risk of soda-lime glass depends strongly on particle size, finely ground glass tending to react as a pozzolan whereas coarser particles can cause expansion [10]. Confining the glass to the 0–150 µm fraction is favorable in this respect, but expansion testing according to GB/T 14684-2022 [24] is required before field use. Third, the scope of application must match the available evidence. The 28-day strengths predicted for the candidate, about 41 MPa in compression and 8 MPa in flexure under the ISO mortar method, are above the strength levels usually specified for masonry and plastering mortars, which are therefore the most direct applications together with precast non-load-bearing units. Use in exposed or structural elements requires the durability data listed in Section 3.5.

### 3.5. Engineering Implications and Limitations

The results of this study have several practical implications for sustainable mortar production. The demonstration that aggregate gradation profoundly modulates WGP effectiveness provides a scientific basis for specifying recycled-glass content in conjunction with aggregate grading curves, rather than as a fixed percentage independent of local aggregate characteristics. In regions where natural sand gradation varies geographically or seasonally—as is common in Chinese construction practice—a single WGP replacement ratio may produce substantially different outcomes depending on the sand source. The present framework allows the gradation–WGP interaction to be quantified and exploited for design advantage rather than treated as an uncontrolled source of variability.

From a practical standpoint, the XGBoost-PSO workflow can in principle be retrained on site-specific experimental datasets and used interactively to generate optimized mix proportions, reducing the number of trial batches required for product qualification. The explicit incorporation of physical constraints—aggregate fraction sum, minimum fraction bounds, flexural strength threshold—ensures that optimization outputs are engineering admissible rather than merely mathematically feasible. This positions the framework as a practical tool for mortar designers operating under circular-economy mandates that require demonstrable recycled material content.

Nevertheless, several limitations restrict the immediate applicability of these results and define the scope of future research. First, the experimental program used a single cement source, a single soda-lime glass composition, and a fixed water-to-cement ratio of 0.50. Variability in cement chemistry, glass cullet origin, color, and alkali content, and admixture use may shift the optimum WGP dosage and gradation. Second, only compressive and flexural strength up to 28 days were assessed. Durability properties including alkali–silica reaction, carbonation resistance, chloride penetration, sulfate attack, and freeze–thaw performance may be affected differently by WGP–aggregate interactions and are critical for structural applications. Third, the data-driven model was trained on 60 experimental observations supplemented by constrained interpolation samples and should be regarded as an interpolation-oriented engineering tool bounded by the tested composition space. Fourth, the optimized mixture identified by XGBoost-PSO lies outside the tested composition domain, and its confirmation follows the program described in Section 3.4. Fifth, the economic and logistical feasibility of size-segregated aggregate blending at industrial scale—which requires sieving and controlled recombination of sand fractions—has not been assessed and warrants a dedicated cost–benefit analysis that also accounts for the energy required to mill WGP to the target fineness.

### 3.6. Scientific Contribution and Mechanistic Summary

The principal contribution is the identification of aggregate packing–WGP synergy as the controlling design concept. At the particle scale, WGP fills interstitial voids; at the hydration scale, reactive silica contributes secondary C-S-H; at the pore scale, suitable gradation and hydration refine pore connectivity; and at the engineering scale, these changes improve compressive and flexural response. The granulometric descriptor analysis (Table 1(b)) provides a quantitative bridge between gradation design and packing geometry, facilitating comparison across mix series and future studies. The results therefore move beyond replacement-ratio optimization toward coordinated multiscale mixture design.

## 4. Conclusions

The following conclusions are drawn from the experimental, microstructural, and data-driven analyses:

(1) WGP dosage and fine-aggregate gradation act synergistically. The best experimental combination (GCS10-M3) increased 28-day compressive and flexural strengths by 14.0% and 42.9%, respectively, demonstrating that WGP performance cannot be optimized independently of aggregate packing. The granulometric descriptor analysis confirms that M3 (FM = 2.50, Cu = 4.7) provides the most favorable void geometry for WGP accommodation.
(2) SEM and MIP show that moderate WGP improves performance through micro-filling, secondary C-S-H formation, pore refinement, and apparent ITZ densification. Excessive WGP combined with an overly fine skeleton (GCS12-F) produces counter-productive porosity increase through dilution and incomplete hydration—an outcome with clear design-rule implications. The chemical components of the proposed mechanisms (pozzolanic reaction extent, portlandite consumption) require confirmation by XRD or TGA analysis in future research.
(3) XGBoost achieved the highest predictive accuracy (R^2^ = 0.9346, RMSE = 0.9860 MPa, MAE = 0.6943 MPa), outperforming RF, LR, and SVR. Sensitivity analysis confirms that the augmentation procedure improved numerical smoothness without introducing systematic bias. The model is an interpolation-oriented surrogate bounded by the tested composition space.
(4) XGBoost-PSO identified a theoretical optimum containing 14.86% WGP in a fines-rich bimodal gradation, with predicted 28-day compressive and flexural strengths of 41.10 MPa and 8.23 MPa. The predicted compressive strength differs from the measured strength of the best tested mixture by less than the model error, whereas the glass content is about 1.5 times higher, so the value of the candidate lies in resource efficiency rather than in strength gain. As the candidate lies outside the tested domain, it is being verified through bracketing mixtures together with flow and pore-structure measurements, and ASR testing is required before field use.

## Figures and Tables

**Table 1:** Mass fractions (wt.%) of the five fine-aggregate gradations. (**a**) Gradation labels: F = fine; M1, M2, M3 = medium gradations of increasing coarseness (M1 < M2 < M3); C = coarse. Each column sums to 100%. (**b**) Granulometric descriptors of the five fine-aggregate gradations. FM: fineness modulus computed as the sum of cumulative percentage retained on the 150, 300, 600, 1180, and 2360 µm sieves divided by 100. Characteristic diameters (D_10_, D_30_, D_50_, D_60_, D_90_) were determined by linear interpolation of the cumulative passing curve derived from the five-fraction mass data in Table 1(a). Cu = D_60_/D_10_; Cc = D_30_^2^/(D_10_ × D_60_). Cu > 4 and 1 < Cc < 3 are conventional thresholds for well-graded sand.

(**a**)					
**Size Range**	**F (%)**	**M1 (%)**	**M2 (%)**	**M3 (%)**	**C (%)**
0–150 µm	10	5	5	5	5
150–300 µm	30	30	20	20	5
300–600 µm	30	30	35	20	15
600–1180 µm	20	15	25	30	25
1180–2360 µm	10	20	15	25	50
(**b**)					
**Parameter**	**F**	**M1**	**M2**	**M3**	**C**
Fineness modulus (FM)	1.90	2.15	2.25	2.50	3.10
D_10_ (µm)	150	175	188	188	300
D_30_ (µm)	250	275	343	375	716
D_50_ (µm)	400	450	510	700	1180
D_60_ (µm)	500	550	600	890	1416
D_90_ (µm)	1180	1770	1573	1888	2124
Uniformity coefficient, Cu	3.3	3.1	3.2	4.7	4.7
Curvature coefficient, Cc	0.83	0.79	1.04	0.84	1.21

**Table 2:** Mixture proportions of WGP-modified cement mortars. The WGP and sand subcolumns report the mass (g) of each particle-size fraction per mortar batch (total aggregate = 1200 g). The weight fraction (wt.%) of each aggregate or WGP size fraction relative to the total aggregate mass can be obtained directly from Table 1(a) for the sand component; WGP fractions replace the corresponding finest sand fractions at the stated substitution levels. All mixtures contained 400 g cement and 200 g water (water-to-cement ratio = 0.50).

ID	Cement (g)	Waste Glass Powder (g)			Sand (g)					Water (g)
		0–150 µm	150–300 µm	300–600 µm	0–150 µm	150–300 µm	300–600 µm	600–1180 µm	1180–2360 µm	
CS-F	400	0	0	0	120	360	360	240	120	200
CS-M1	400	0	0	0	60	360	360	180	240	200
CS-M2	400	0	0	0	60	240	420	300	180	200
CS-M3	400	0	0	0	60	240	240	360	300	200
CS-C	400	0	0	0	60	60	180	300	600	200
GCS8-F	400	96	0	0	24	360	360	240	120	200
GCS8-M1	400	60	36	0	0	324	360	180	240	200
GCS8-M2	400	60	36	0	0	204	420	300	180	200
GCS8-M3	400	60	36	0	0	204	240	360	300	200
GCS8-C	400	60	36	0	0	24	180	300	600	200
GCS10-F	400	120	0	0	0	360	360	240	120	200
GCS10-M1	400	60	60	0	0	300	360	180	240	200
GCS10-M2	400	60	60	0	0	180	420	300	180	200
GCS10-M3	400	60	60	0	0	180	240	360	300	200
GCS10-C	400	60	60	0	0	0	180	300	600	200
GCS12-F	400	120	24	0	0	336	360	240	120	200
GCS12-M1	400	60	84	0	0	276	360	180	240	200
GCS12-M2	400	60	84	0	0	156	420	300	180	200
GCS12-M3	400	60	84	0	0	156	240	360	300	200
GCS12-C	400	60	60	24	0	0	156	300	600	200

**Table 3:** MIP-derived pore-structure parameters of the six selected mortars.

Mix ID	Median Pore Diameter (Volume)/nm	Median Pore Diameter (Area)/nm	Meam Pore Diameter (4V/A) nm	Total Porosity/%
CS-F	74.0	14.7	38.6	12.8
CS-M3	52.6	15.8	30.3	11.8
CS-C	46.8	13.7	27.6	8.7
GCS12-F	137.3	13.1	42.5	13.7
GCS8-M3	55.6	12.9	29.8	9.8
GCS10-C	62.0	11.8	31.8	10.2

**Table 5:** Optimal mixture identified by the XGBoost-PSO framework.

Parameter	Symbol	Optimized Value
WGP replacement ratio (%)	glass	14.86
Fine aggregate 0–150 µm (%)	s_1_	21.98
Fine aggregate 150–300 µm (%)	s_2_	15.36
Fine aggregate 300–600 µm (%)	s_3_	9.25
Fine aggregate 600–1180 µm (%)	s_4_	30.29
Fine aggregate 1180–2360 µm (%)	s_5_	23.13
Curing age (d)	age	28
Predicted compressive strength (MPa)	f_comp	41.10
Predicted flexural strength (MPa)	f_flex	8.23

## Data Availability

The original contributions presented in this study are included in the article. Further inquiries can be directed to the corresponding author.
